# Vericiguat Therapy Is Associated with Reverse Myocardial Remodeling in Chronic Heart Failure with Reduced Ejection Fraction

**DOI:** 10.3390/jcdd13010017

**Published:** 2025-12-29

**Authors:** Tine Bajec, Neža Žorž, Sabina Ugovšek, Gregor Zemljič, Andraž Cerar, Sabina Frljak, Renata Okrajšek, Petra Girandon Sušanj, Miran Šebeštjen, Bojan Vrtovec, Gregor Poglajen

**Affiliations:** 1 Advanced Heart Failure and Transplantation Center, Department of Cardiology, University Medical Centre Ljubljana, 1000 Ljubljana, Slovenia; 2 Department of Internal Medicine, Faculty of Medicine, University of Ljubljana, 1000 Ljubljana, Slovenia

**Keywords:** soluble guanylate cyclase stimulator, heart failure, myocardial reverse remodeling

## Abstract

Background and aims: Vericiguat lowers cardiovascular death or heart-failure hospitalization in recently worsened heart failure with reduced ejection fraction (HFrEF), but its effects on cardiac remodeling are less well characterized. Our aim was to evaluate whether the addition of vericiguat to guideline-directed medical therapy (GDMT) promotes reverse myocardial remodeling in patients with HFrEF and recent worsening. Methods: We conducted a prospective, non-randomized, single-center study enrolling 34 consecutive patients with HFrEF who had experienced recent worsening and were on stable GDMT for at least 3 months prior to decompensation. Clinical, biochemical, and echocardiographic assessments were performed at baseline and at 6 months. Results: A total of 24 patients completed the 6-month follow-up (mean age 63 ± 9 years; 92% male), 96% of whom were in New York Heart Association (NYHA) class III or IV. After 6 months of vericiguat therapy, right ventricular systolic function improved significantly, with an increase in tricuspid annular plane systolic excursion (TAPSE) from 18.5 ± 4.3 mm to 21.4 ± 4.8 mm (*p* = 0.003). Left ventricular systolic function improved, with a numerical increase in left ventricular ejection fraction (LVEF) (30.1 ± 5.9% to 32.2 ± 10.5%; *p* = 0.122) and a significant increase in left ventricular outflow tract velocity-time integral (LVOT VTI) (14.8 ± 3.7 cm to 16.1 ± 3.8 cm; *p* = 0.011). Functional improvements were accompanied by structural remodeling, including reductions in right ventricular internal diameter in diastole (RVIDd) (40.5 ± 5.8 mm to 37.9 ± 6.9 mm; *p* = 0.002) and left ventricular end-systolic volume (LVESV) (144.0 ± 40.3 mL to 132.4 ± 61.0 mL; *p* = 0.031). N-terminal pro-B-type natriuretic peptide (NT-proBNP) levels also decreased significantly (median 1829.0 ng/mL to 1241.0 ng/mL; *p* = 0.03). Conclusions: In patients with HFrEF and recent worsening, the addition of vericiguat to GDMT may be associated with reverse myocardial remodeling.

## 1. Introduction

Recent years have brought significant advances in the treatment of heart failure with reduced ejection fraction (HFrEF), most notably with the findings from the PARADIGM-HF and DAPA-HF trials, which have redefined guideline-directed medical therapy (GDMT). Current GDMT now includes four foundational drug classes: beta-blockers (BB), angiotensin receptor–neprilysin inhibitors (ARNIs), mineralocorticoid receptor antagonists (MRAs), and sodium–glucose cotransporter 2 inhibitors (SGLT2i) [1,2,3]. While this comprehensive pharmacologic strategy has improved outcomes in HFrEF, a substantial residual risk persists [4]. In the EMPEROR-Reduced trial, most patients receiving empagliflozin were also on optimized GDMT—94.7% on BBs, 88.8% on ACEi/ARB or ARNIs, and 70.1% on MRAs—yet 13.2% experienced worsening heart failure and 10.0% died from cardiovascular causes during follow-up [5]. Similarly, the EMPULSE trial, which enrolled patients with acute de novo or decompensated chronic heart failure, showed that even among those receiving quadruple therapy, 10.6% had worsening heart failure events and 4.2% died within 90 days [6]. These data highlight the continued need for novel therapeutic strategies to address the high risk of morbidity and mortality in the HFrEF patient population, particularly in a subpopulation of patients with recent heart failure decompensation requiring additional interventions [7].

One such potential strategy targets the nitric oxide (NO)–soluble guanylate cyclase (sGC)–cyclic guanosine monophosphate (cGMP) pathway. In HFrEF, elevated oxidative stress and inflammation reduce NO bioavailability, impairing cGMP production and disrupting downstream signaling in endothelial cells, vascular smooth muscle cells, and cardiomyocytes, thereby contributing to both vascular and myocardial dysfunction [8,9,10,11,12]. sGC stimulators, such as vericiguat, enhance cGMP synthesis by sensitizing sGC to endogenous NO and activating it independently of NO, thereby restoring NO–sGC–cGMP signaling under conditions of NO deficiency [13]. A phase 2 trial (SOCRATES-Reduced) suggested that higher doses of vericiguat were associated with greater reductions in NT-proBNP levels [14], and the phase 3 VICTORIA trial later demonstrated that vericiguat significantly reduced the risk of cardiovascular death or hospitalization for heart failure in patients with HFrEF and recent decompensation (35.5% vs. 38.5%, *p* = 0.02) [15]. However, the mechanisms underlying these clinical benefits remain incompletely understood and underexplored.

Preclinical studies indicate that enhancing cGMP signaling can reverse maladaptive myocardial changes by reducing hypertrophy, restoring proteasomal activity, promoting autophagy, and limiting fibrosis, which are hallmarks of reverse remodeling of the failing myocardium [16,17,18,19,20]. Moreover, many therapies shown to improve long-term outcomes in HFrEF were also shown to induce significant reverse myocardial remodeling [21].

Accordingly, we evaluated whether adding vericiguat to contemporary GDMT promotes reverse myocardial remodeling in patients with HFrEF and recent heart failure worsening. Given the established efficacy of sGC stimulation in pulmonary vascular disease and our observations of improved right-sided structure and function in left ventricular assist device (LVAD) recipients initiated on vericiguat, we prespecified right-ventricular remodeling as the primary focus [22].

## 2. Methods

### 2.1. Patient Population

We conducted a single-center, prospective, longitudinal study that included 25 consecutive patients with HFrEF and recent heart failure worsening. Patient enrolment took place between April 2022 and February 2023, with all patients recruited and treated at the Advanced Heart Failure and Transplantation Center, Department of Cardiology, University Medical Centre Ljubljana. The study was conducted in accordance with the principles of the Declaration of Helsinki and was approved by the National Medical Ethics Committee of the Republic of Slovenia (0120-20/2023/3). Written informed consent was obtained from all participants prior to enrolment.

Eligible participants were adults ( ≥18 years) with chronic heart failure (NYHA class II–IV) and a left ventricular ejection fraction (LVEF) ≤40%, who had experienced heart failure worsening within six months prior to enrolment, requiring either hospitalization or outpatient intravenous diuretic therapy. All patients were on optimized GDMT for HFrEF, as recommended by current ESC guidelines, for at least three months before enrolment [23]. Exclusion criteria were: symptomatic hypotension or systolic blood pressure <100 mmHg; current use of long-acting nitrates, sGC stimulators, or phosphodiesterase type 5 inhibitors; known hypersensitivity to sGC stimulators; long-term mechanical circulatory support; hemodynamic instability requiring inotropic or vasopressor support; active infection requiring antibiotic or antiviral therapy; multiorgan failure; chronic kidney disease stage 4 or 5; and chronic liver disease classified as Child–Pugh class B or C.

### 2.2. Study Design

**Figure 1:**
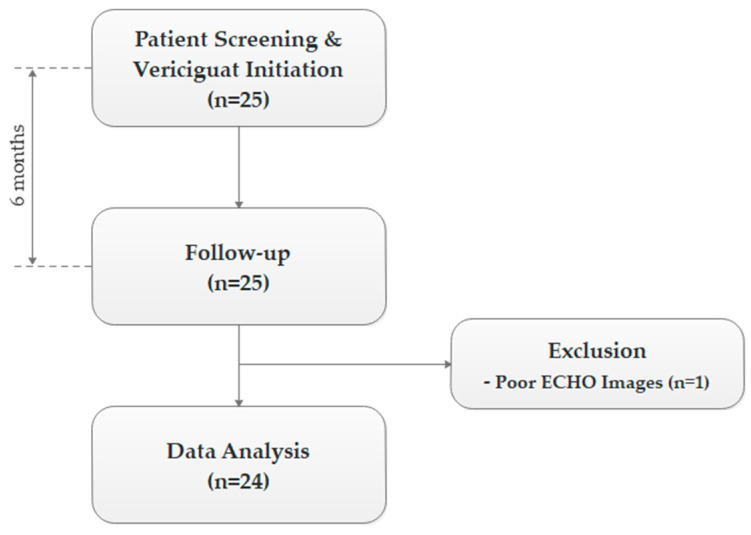
Study Design.

We screened patients at the time of hospital admission or during an unplanned outpatient visit. After achieving clinical stabilization, we initiated vericiguat in those who met the inclusion criteria. Treatment started at 2.5 mg and was up-titrated to 5 mg and then to 10 mg at two-week intervals. If patients developed symptomatic hypotension or their systolic blood pressure dropped below 100 mmHg, we adjusted the dose of vericiguat accordingly. Background HF therapy, including diuretic dosing, was kept unchanged throughout the study period. We performed clinical examination, hemodynamic assessment, NYHA functional class evaluation, ECG, biochemical testing, and transthoracic echocardiography at baseline and again at the 6-month follow-up (Figure 1).

### 2.3. Biochemical Evaluation

We collected venous blood samples at baseline and at the 6-month follow-up, following standard laboratory operating procedures. The Institute of Clinical Chemistry and Biochemistry at the University Medical Centre Ljubljana conducted routine biochemical and haematological analyses. Measured parameters included electrolytes, complete blood count, renal and liver function tests, and plasma NT-proBNP levels. Laboratory personnel who analyzed the samples had access to the patients’ clinical data and were not blinded.

### 2.4. Echocardiographic Imaging

We performed transthoracic echocardiographic examinations at baseline and at the 6-month follow-up using a GE Vivid E9 ultrasound system (Vingmed Ultrasound, Horten, Norway) equipped with a 2.5 MHz transducer. Recordings were obtained in the parasternal long-axis (PLAX), parasternal short-axis (PSAX), apical four-chamber (A4C), apical two-chamber (A2C), and apical long-axis views, following current recommendations from the ESC and ASE [24]. The echocardiographic assessment included measurements of left ventricular ejection fraction (LVEF), left ventricular end-systolic dimension (LVESD), end-diastolic dimension (LVEDD), end-systolic volume (LVESV), and end-diastolic volume (LVEDV). We also measured left ventricular outflow tract velocity time integral (LVOT VTI), the E/e′ ratio (transmitral E wave velocity to tissue Doppler e′ wave velocity), tricuspid annular plane systolic excursion (TAPSE), fractional area change (FAC), tricuspid lateral annular systolic velocity (S′), tricuspid regurgitation gradient (TRgrad), and right ventricular inflow tract diameter in diastole (RVIDd). We assessed left ventricular size in the PLAX, A4C, and A2C views. Global left ventricular systolic function was evaluated using the Simpson biplane method, while TAPSE served as an index of right ventricular systolic function. All measurements were averaged from three consecutive cardiac cycles and analyzed using EchoPAC software (version 1.13).

### 2.5. Outcomes

We defined the primary outcome as the absolute change in right ventricular systolic function parameters (TAPSE, tricuspid annular S′, and FAC) from baseline to the 6-month follow-up.

Secondary outcomes included absolute changes in left ventricular systolic function (LVEF) and dimensions (LVESD and LVEDD), left ventricular volumes (LVESV and LVEDV), right ventricular dimensions (RVIDd), stroke volume (LVOT VTI), left ventricular diastolic function (E/e′), tricuspid valve gradient (TRgrad), NYHA functional class, and plasma NT-proBNP levels between baseline and the 6-month follow-up.

### 2.6. Statistical Analysis

We assessed the distribution of paired differences (follow-up minus baseline) with the Shapiro–Wilk test and quantile–quantile plots. Normally distributed continuous variables are presented as mean ± standard deviation (SD), while skewed data are reported as median (interquartile range). Categorical variables are presented as counts (percentages).

Changes from baseline to 6 months were analyzed using paired tests: the paired *t*-test for approximately normally distributed paired differences and the Wilcoxon signed-rank test otherwise. For normally distributed paired differences, results are reported as mean change with 95% CI; for non-normally distributed paired differences, results are reported as median change (interquartile range). All tests were two-tailed, with *p* < 0.05 considered statistically significant. Analyses were performed using IBM SPSS ver. 25.0 (Armonk, NY, USA).

Sample size calculation: the sample size was based on the primary endpoint of within-patient change in TAPSE from baseline to 6 months. Assuming a mean paired difference of 2.5 mm (SD 4 mm), two-sided α = 0.05, and 80% power, the required sample size was *n* = 21. Because this was a high-risk cohort, we intentionally over-enrolled to offset expected dropout due to escalation to advanced HF therapies. Assumptions for the mean change and variability were informed by TAPSE changes and dispersion in recently worsened HFrEF cohorts on contemporary GDMT [25,26].

## 3. Results

### 3.1. Patient Data

Of the 25 eligible patients that were enrolled in the study, we excluded one patient due to insufficient echocardiographic image quality, leaving 24 patients eligible for final statistical analysis (Figure 1). Detailed baseline patient characteristics are presented in Table 1.

The study population was predominantly male (92%) with a mean age of 62.8 years. The most common cause of heart failure was non-ischemic dilated cardiomyopathy (66.7%), followed by ischemic heart disease (33.3%). At baseline, patients showed severe functional limitation (95.9% in NYHA class III or IV), with a mean LVEF of 30.1% and a median plasma NT-proBNP level of 1829.0 ng/mL. The most prevalent comorbidities included arterial hypertension, atrial fibrillation, diabetes mellitus, and hyperlipidemia. Most patients were receiving heart failure guideline-directed medical therapy (ACEi/ARB/ARNI 100%, BB 96%, MRA 100%, and SGLT2i 96%), and 60% were receiving loop diuretic treatment.

### 3.2. Primary Outcome

**Figure 2:**
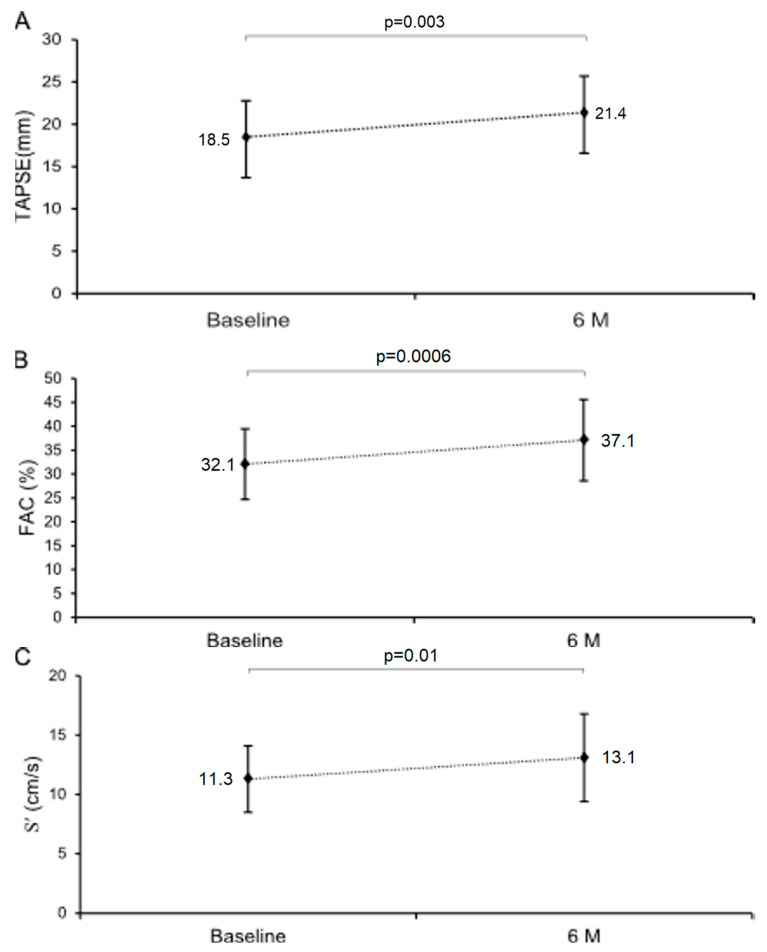
Effect of vericiguat on right ventricular systolic function: TAPSE (**A**), FAC (**B**), S′ (**C**). Abbreviations: TAPSE, tricuspid annular plane systolic excursion; FAC, fractional area change; S′, tricuspid lateral annular systolic velocity.

At 6 months follow-up, vericiguat therapy was associated with improved right-ventricular systolic function, with increases in TAPSE of +2.8 ± 4.2 mm (95% CI: 1.1 to 4.6; *p* = 0.003; Figure 2), tricuspid annular S′ of +1.8 ± 3.0 cm/s (95% CI: 0.4 to 3.2; *p* = 0.01), and FAC of +5.0 ± 6.0% (95% CI: 2.4 to 7.6; *p* = 0.0006).

### 3.3. Secondary Outcomes

#### 3.3.1. Myocardial Function

**Figure 3:**
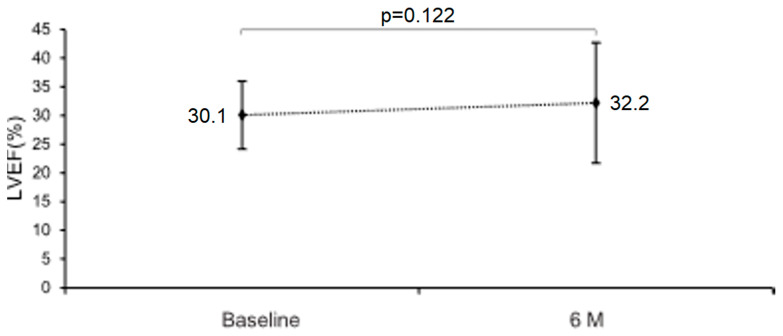
Effect of vericiguat on left ventricular systolic function. Abbreviations: LVEF, left ventricular ejection fraction.

Vericiguat therapy was associated with a numerical, but statistically non-significant increase in LVEF of +2.1 ± 6.4% (95% CI: −0.6 to 4.8; *p* = 0.122; Figure 3). However, we found a significant increase in LVOT VTI (Table 2). No significant changes were observed in left ventricular diastolic function and pulmonary hemodynamics (Table 2).

#### 3.3.2. Myocardial Structure

Structural indices were consistent with biventricular reverse remodeling. At 6 months, LVESV decreased significantly, whereas there was a numerical decrease in LVEDV that did not reach statistical significance (Table 2). Additionally, no significant changes were observed in left ventricular dimensions (LVEDD, LVESD; Table 2). Importantly, we additionally established a significant effect of vericiguat therapy on right ventricular reverse remodeling, as a significant reduction in RVIDd was observed at 6 months follow-up (Table 2).

#### 3.3.3. Neurohumoral Activation and NYHA Functional Class

**Figure 4:**
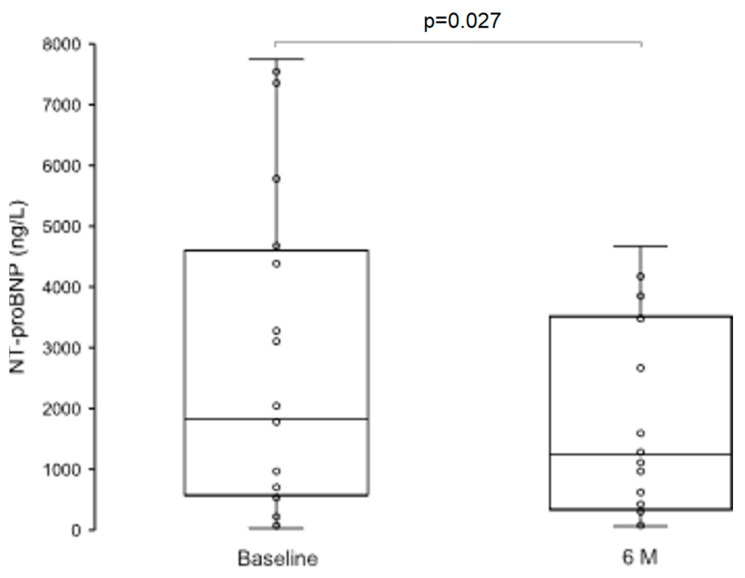
Effect of vericiguat on plasma NT-proBNP levels. Abbreviations: NT-proBNP, N-terminal pro–B-type natriuretic peptide.

**Figure 5:**
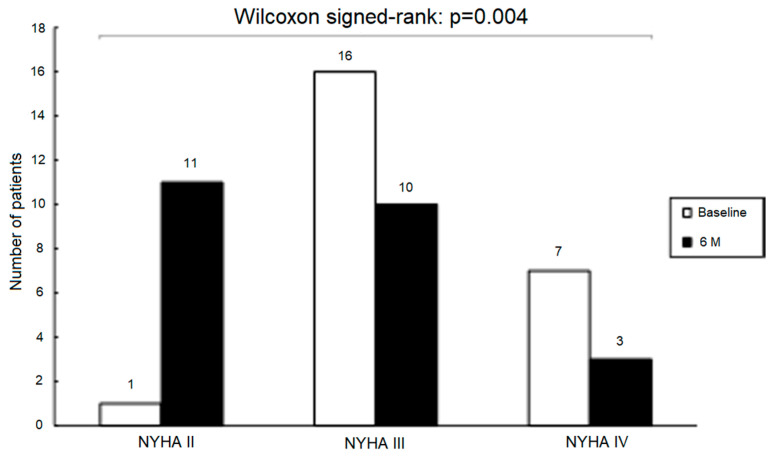
Distribution of patients across NYHA functional classes at baseline and at 6 months. Abbreviations: NYHA, New York Heart Association.

Vericiguat use was associated with a significant reduction in plasma NT-proBNP from 1829.0 (658.3–4457.3) ng/mL at baseline to 1240.8 (396.0–3489.4) ng/mL at 6 months (*p* = 0.027, Figure 4). Additionally, we found significant improvement of the NYHA functional class at 6-month follow-up (Figure 5).

## 4. Discussion

This study is among the first to provide detailed echocardiographic evidence on the effects of vericiguat therapy on myocardial structure and function in patients with HFrEF and recent heart failure worsening. Our findings suggest that the addition of vericiguat to optimized GDMT may improve right ventricular systolic function, supported by concordant increases in TAPSE, tricuspid annular S′, and RV FAC, and may also support left ventricular functional recovery, although changes in LVEF did not reach statistical significance. Importantly, we observed favorable structural changes—including reductions in LVESV, RVIDd, and a trend toward reduced LVEDV—indicating the potential for reverse structural remodeling of the failing myocardium. These functional and structural improvements were accompanied by a significant reduction in NT-proBNP levels and an improvement in NYHA functional class, supporting the hypothesis that vericiguat may contribute to symptomatic and hemodynamic recovery in this high-risk population.

cGMP is a pleiotropic second messenger that is central to myocardial and vascular homeostasis. In HFrEF, NO–sGC–cGMP signaling is impaired due to reduced NO bioavailability, oxidative stress–related blunting of sGC responsiveness, and increased cGMP degradation by phosphodiesterases, resulting in a net cGMP deficit. Reduced downstream cGMP/PKG signaling is associated with loss of protective restraint on maladaptive pathways implicated in hypertrophy, fibrosis, inflammation, and vascular dysfunction. Vericiguat, an sGC stimulator, increases cGMP by both sensitizing sGC to endogenous NO and directly stimulating sGC activity, thereby augmenting cGMP generation even when NO signaling is compromised. In preclinical models, enhancement of NO–sGC–cGMP signaling has been linked to anti-hypertrophic, anti-fibrotic, and anti-inflammatory effects and attenuation of adverse remodeling, supporting biological plausibility for beneficial structural and functional effects in humans, although the precise mechanisms in patients remain to be established [20,27,28].

The most robust signal of vericiguat efficacy in our cohort was a significant improvement in RV systolic function across indices (TAPSE, tricuspid annular S′, and FAC). This improvement suggests that sGC stimulation may favorably affect the contractility of the right ventricle, which is especially vulnerable to pressure overload and systemic congestion in advanced heart failure. Additionally, right ventricular (dys)function strongly predicts clinical outcomes in this patient cohort [29]. Given that right ventricular function is rarely successfully targeted by current heart failure therapies, this observation is of particular interest.

In contrast, the change in LVEF did not reach statistical significance and should therefore be interpreted cautiously. Although LVEF in our cohort showed a numerically favorable trend (+2.1 ± 6.4%), the magnitude of change was small. Nevertheless, the directionality of change aligns with the hypothesized beneficial effect of vericiguat on myocardial contractility through enhancement of the NO–sGC–cGMP pathway. Preclinical evidence supports this mechanism, showing that increased cGMP levels improve sarcomere function, modulate calcium handling, and reverse maladaptive myocardial hypertrophy [18,30,31,32,33,34].

Despite improvements in systolic measures of the failing myocardium, vericiguat did not significantly alter indices of diastolic function in our study. Specifically, the E/e′ ratio—a widely used surrogate of LV filling pressure—remained largely unchanged over the 6-month follow-up. This may reflect a “ceiling effect,” as baseline diastolic dysfunction was only mildly elevated (mean E/e′ of 14.1), providing limited opportunity for improvement. Alternatively, the timeline of the study may have been too short to capture slower changes in myocardial compliance or fibrosis resolution, key determinants that underlie the diastolic function of the myocardium. Mechanistically, protein kinase G mediated phosphorylation of titin and troponin I has been shown to enhance diastolic relaxation and reduce stiffness in preclinical models [35,36]. The absence of such an effect in our study may suggest that vericiguat’s influence on diastolic function is more variable, delayed, or dependent on the degree of pre-existing diastolic dysfunction. Further investigation in patients with more pronounced diastolic abnormalities is warranted.

Our findings also demonstrated significant reductions in LVESV and RVIDd, as well as a trend toward a reduction in LVEDV, supporting the potential of vericiguat to induce reverse myocardial remodeling. These changes are consistent with favorable shifts in ventricular loading conditions and myocardial geometry. Importantly, reductions in ventricular volumes often precede and predict improvements in systolic performance and long-term prognosis of heart failure patients [37]. The decrease in LVESV is particularly notable, as it correlates strongly with improved survival in HFrEF and reflects a fundamental reversal of adverse myocardial remodeling. Although not statistically significant, the observed reduction in LVEDV further supports this interpretation. The reduction in RVIDd also aligns with the observed improvement in RV systolic function, suggesting that vericiguat may favorably influence both right ventricular structure and function—possibly through reductions in pulmonary pressures, afterload, or intrinsic right heart remodeling. These findings are in line with published preclinical data demonstrating that sGC stimulation reverses hypertrophy and fibrosis, normalizes myocardial heart failure-associated gene expression, and improves ventricular geometry [32].

Clinical evidence on vericiguat’s effects on cardiac structure and function is mixed. In the VICTORIA echocardiographic substudy, both vericiguat and placebo groups showed small within-group changes over approximately 8 months, but between-group differences in myocardial structure and function were not significant, suggesting no incremental reverse-remodeling signal beyond background therapy during follow-up [25]. Several factors may explain the observed discrepancies. Compared with our cohort, the VICTORIA population was older, more comorbid, and more decompensated (median baseline NT-proBNP, 2591.0 vs. 1829.0 ng/mL), reflecting a more advanced disease stage with limited potential for measurable remodeling. Consistent with a severity gradient, subanalyses of the VICTORIA trial indicate greater clinical benefit at lower baseline NT-proBNP concentrations, with their attenuation at the highest levels of NT-proBNP [38]. Differences in contemporary background therapy may also have contributed. Compared with VICTORIA, our cohort had broader use of ARNIs (96% vs. 16%) and SGLT2 inhibitors (96% vs. 2.4%)—agents that augment particulate guanylate cyclase-mediated cGMP signaling and exert anti-inflammatory and antioxidant effects—which may have complemented sGC stimulation and fostered a milieu more favorable to NO–sGC–cGMP coupling [39,40,41,42,43].

Smaller cohorts of patients with recently worsened HFrEF on GDMT generally report more favorable reverse-remodeling patterns. Zhan et al. studied a younger, less decompensated cohort (average baseline NT-proBNP 563.0 ng/mL) and observed improvements in LVEF and left-sided dimensions, with a reduction in right-ventricular diameter over 6 months [26]. Their change in TAPSE was not statistically significant (*p* = 0.054), in contrast to our increase, which may reflect more advanced right ventricular dysfunction in that cohort (baseline TAPSE: 15.0 vs. 18.5 mm) and a slower remodeling trajectory requiring longer follow-up to detect. By contrast, Tian et al. enrolled a larger but less well-compensated cohort—nearly half had baseline NT-proBNP >1.000 ng/mL—and did not observe a significant reverse-remodeling signal with add-on vericiguat versus GDMT [44].

Lower oxidative stress provides a mechanistic rationale for the more pronounced reverse remodeling and greater clinical benefit seen in better-compensated HFrEF. In states of heightened oxidative stress, the heme iron within sGC is oxidized from ferrous (Fe^2+^) to ferric (Fe^3+^), leading to heme dissociation, reduced NO binding, and diminished responsiveness to sGC stimulators such as vericiguat [45,46,47]. Even though the VICTOR trial failed to meet its primary outcome, vericiguat use in stable, ambulatory HFrEF was associated with significant reductions in cardiovascular and all-cause mortality [48]. Whether such clinical signals are accompanied by structural reverse remodeling remains uncertain. Overall, the available evidence supports a context-dependent remodeling effect that appears more likely in less decompensated patients and may require adequate time, optimized background therapy, and preserved NO–sGC coupling to emerge.

Improvements in myocardial structure and function in our cohort were accompanied by meaningful clinical benefits and a reduction in neurohumoral activation. NYHA functional class improved in the majority of patients, and plasma NT-proBNP levels decreased significantly. These changes reflect both improved hemodynamic stability and reduced myocardial wall stress, supporting the therapeutic value of vericiguat in symptomatic relief and neurohumoral modulation. NT-proBNP is not only a marker of ventricular stretch but also a prognostic biomarker used to guide therapy. Reductions in NT-proBNP after treatment correlate with improved outcomes across various heart failure interventions [49]. Notably, our findings contrast with those from the VICTORIA substudy, where NT-proBNP reductions were modest and not clearly associated with reverse remodeling. As previously discussed, differences in patient stability and background therapy may have contributed to these divergences.

### Study Limitations

This study has several limitations that need to be acknowledged. The small sample size and single-center design limit generalizability and reduce statistical power, particularly for detecting more subtle effects or subgroup interactions. Although patients were enrolled consecutively and follow-up was complete, reducing the likelihood of selective inclusion, the single-arm design without a contemporaneous control group precludes causal inference. Accordingly, the observed changes may partially reflect recovery following decompensation and/or regression to the mean (i.e., improvement after an unusually adverse baseline measurement due to natural variability). Importantly, background GDMT remained unchanged during the study, minimizing confounding from concomitant medication adjustments; however, the highly optimized baseline therapy and specialized-care setting may limit external validity to less-optimized populations. Furthermore, the follow-up period of 6 months may not be sufficient to fully capture the time course of reverse remodeling or long-term outcomes. Additionally, echocardiographic evaluation of heart morphology and function has inherent limitations, particularly in the right ventricle, where assessment of both size and function can be challenging due to complex geometry and position of the right ventricle. Future studies should utilize cardiac MRI to more comprehensively and accurately assess the reverse remodeling of the right ventricle. Despite these limitations, our study represents an important early exploration of vericiguat’s structural, functional, and symptomatic effects in real-world HFrEF patients. The findings provide a strong rationale for further controlled studies focusing on patient selection, mechanistic pathways, and combination therapy strategies to optimize the use of sGC stimulators in advanced heart failure care.

## 5. Conclusions

Our findings suggest that adding vericiguat to optimized GDMT in patients with HFrEF and recent heart failure worsening may promote reverse cardiac remodeling, with improvements in left and right ventricular function, structure, NT-proBNP levels, and NYHA class. These results support the hypothesis that reverse remodeling may contribute to the clinical benefits observed in trials of vericiguat. Larger, controlled studies in stable HFrEF populations on contemporary GDMT are needed to confirm these effects and explore potential synergistic effects between vericiguat and ARNIs and SGLT2 inhibitors.

## Figures and Tables

**Table 1:** Baseline Characteristics of the Study Population.

Variable	Study Population (*n* = 24)
Age (years), mean (SD)	62.8 (9.3)
Gender (male)	22 (91.7%)
NYHA functional class	
II	1 (4.2%)
III	16 (66.7%)
IV	7 (29.2%)
Etiology of heart failure	
DCMP	16 (66.7%)
IHD	8 (33.3%)
Laboratory measurements, mean (SD)	
Sodium (mmol/L)	139.7 (3.5)
Potassium (mmol/L)	4.7 (0.5)
Creatinine (umol/L)	103.4 (23.2)
eGFR (CKD-EPI)/1.73 m^2^	62.0 (16.2)
Bilirubin (umol/L)	16.2 (12.0)
AST (ukat/L)	0.5 (0.2)
ALT (ukat/L)	0.5 (0.2)
gGT (ukat/L)	1.2 (1.1)
NT-proBNP (ng/mL), median (IQR)	1829.0 (658.3–4457.3)
Hemoglobin (g/L)	152.3 (19.9)
Leukocytes (10^9^/L)	8.0 (2.3)
Platelet count (10^9^/L)	195.2 (48.2)
Comorbidities	
Diabetes mellitus	9 (37.5%)
Arterial hypertension	14 (58.3%)
COPD	1 (4.2%)
Atrial fibrillation	11 (45.8%)
Chronic kidney disease	6 (25.0%)
Hyperlipidemia	9 (37.5%)
Medications	
ARNI	23 (95.8%)
ACEi/ARB	1 (4.2%)
BB	23 (95.8%)
MRA	24 (100%)
SGLT2i	23 (95.8%)
Digitalis	3 (12.5%)
Diuretics	14 (58.3%)
Statins	14 (58.3%)
Oral anticoagulant	11 (45.8%)
Acetylsalicylic acid	5 (20.8%)

Continuous variables are presented as mean (standard deviation) or median (interquartile range), as appropriate. Categorical variables are expressed as count (percentage). Abbreviations: ACEi/ARB, angiotensin-converting enzyme inhibitor/angiotensin II receptor blocker; ALT, alanine transaminase; ARNI, angiotensin receptor–neprilysin inhibitor; AST, aspartate transaminase; BB, beta-blocker; COPD, chronic obstructive pulmonary disease; DCMP, dilated cardiomyopathy; eGFR, estimated glomerular filtration rate; gGT, γ-glutamyl transpeptidase; IHD, ischemic heart disease; IQR, interquartile range; MRA, mineralocorticoid receptor antagonist; NT-proBNP, N-terminal pro–B-type natriuretic peptide; NYHA, New York Heart Association; SD, standard deviation; SGLT2i, sodium–glucose cotransporter-2 inhibitor.

**Table 2:** Changes in Secondary Echocardiographic Parameters.

Variable	Baseline *n* = 24	6M *n* = 24	Change	*p*-Value
LVOT VTI (cm)	14.8 ± 3.7	16.1 ± 3.8	1.6 ± 2.7	0.011
LVEDV (ml)	213.3 ± 54.9	197.6 ± 65.6	−15.7 ± 37.4	0.051
LVESV (ml)	149.5 (112.5–175.5)	129.0 (79.5–166.5)	−14.5 (−36.0–4.0)	0.031
LVEDD (mm)	63.8 ± 5.9	62.8 ± 7.0	−1.0 ± 4.7	0.328
LVESD (mm)	55.3 ± 6.0	53.5 ± 8.4	−1.8 ± 5.7	0.145
E/e′	14.1 ± 5.1	15.3 ± 7.3	1.0 ± 6.0	0.473
RVIDd (mm)	40.5 ± 5.8	37.9 ± 7.0	−2.3 ± 3.8	0.002
TRgrad (mmHg)	34.9 ± 9.9	33.2 ± 10.1	−1.7 ± 5.3	0.130

Changes in secondary echocardiographic parameters after 6 months of vericiguat therapy. Continuous variables are presented as mean (standard deviation) or median (interquartile range), as appropriate. Abbreviations: E/e′, ratio of early mitral inflow velocity to mitral annular early diastolic velocity; LVEDD, left ventricular end-diastolic dimension; LVEDV, left ventricular end-diastolic volume; LVESD, left ventricular end-systolic dimension; LVESV, left ventricular end-systolic volume; LVOT VTI, left ventricular outflow tract velocity–time integral; RVIDd, right ventricular internal diameter in diastole; TRgrad, tricuspid regurgitation pressure gradient.

## Data Availability

The data presented in this study are available from the corresponding author upon reasonable request due to applicable privacy and institutional confidentiality restrictions.

## Abbreviations

The following abbreviations are used in this manuscript:

A2C	apical 2-chamber view
A4C	apical 4-chamber view
ACEi	angiotensin-converting enzyme inhibitor(s)
ARB	angiotensin receptor blocker(s)
ARNI	angiotensin receptor–neprilysin inhibitor
BB	beta-blockers
cGMP	cyclic guanosine monophosphate
E/e′	ratio of early mitral inflow velocity to mitral annular early diastolic velocity
FAC	fractional area change
GDMT	guideline-directed medical therapy
HFrEF	heart failure with reduced ejection fraction
LVAD	left ventricular assist device
LVEDV	left ventricular end-diastolic volume
LVEDD	left ventricular end-diastolic dimension
LVEF	left ventricular ejection fraction
LVESD	left ventricular end-systolic dimension
LVESV	left ventricular end-systolic volume
LVOT VTI	left ventricular outflow tract velocity-time integral
MRA	mineralocorticoid receptor antagonist(s)
NO	nitric oxide
NYHA	New York Heart Association
PLAX	parasternal long-axis
PSAX	parasternal short-axis
RVIDd	right ventricular internal diameter in diastole
sGC	soluble guanylate cyclase
SGLT2i	sodium-glucose cotransporter-2 inhibitor(s)
TAPSE	tricuspid annular plane systolic excursion
TRgrad	tricuspid regurgitation pressure gradient
S′	tricuspid lateral annular systolic velocity
